# Predicting human cardiac QT alterations and pro-arrhythmic effects of compounds with a 3D beating heart-on-chip platform

**DOI:** 10.1093/toxsci/kfac108

**Published:** 2022-10-13

**Authors:** Roberta Visone, Ferran Lozano-Juan, Simona Marzorati, Massimo Walter Rivolta, Enrico Pesenti, Alberto Redaelli, Roberto Sassi, Marco Rasponi, Paola Occhetta

**Affiliations:** Department of Electronics, Information and Bioengineering, Politecnico di Milano, Milan, 20133, Italy; BiomimX Srl, Milan, 20158, Italy; Department of Electronics, Information and Bioengineering, Politecnico di Milano, Milan, 20133, Italy; BiomimX Srl, Milan, 20158, Italy; Accelera Srl, Milan, 20014, Italy; Department of Computer Science, Università degli Studi di Milano, Milan, 20133, Italy; Accelera Srl, Milan, 20014, Italy; Department of Electronics, Information and Bioengineering, Politecnico di Milano, Milan, 20133, Italy; Department of Computer Science, Università degli Studi di Milano, Milan, 20133, Italy; Department of Electronics, Information and Bioengineering, Politecnico di Milano, Milan, 20133, Italy; Department of Electronics, Information and Bioengineering, Politecnico di Milano, Milan, 20133, Italy; BiomimX Srl, Milan, 20158, Italy

**Keywords:** QT prolongation, cardiac toxicity, organs-on-chip, arrhythmias, *in vitro* cardiac model, electrophysiology

## Abstract

Determining the potential cardiotoxicity and pro-arrhythmic effects of drug candidates remains one of the most relevant issues in the drug development pipeline (DDP). New methods enabling to perform more representative preclinical *in vitro* studies by exploiting induced pluripotent stem cell-derived cardiomyocytes (iPSC-CM) are under investigation to increase the translational power of the outcomes. Here we present a pharmacological campaign conducted to evaluate the drug-induced QT alterations and arrhythmic events on uHeart, a 3D miniaturized *in vitro* model of human myocardium encompassing iPSC-CM and dermal fibroblasts embedded in fibrin. uHeart was mechanically trained resulting in synchronously beating cardiac microtissues in 1 week, characterized by a clear field potential (FP) signal that was recorded by means of an integrated electrical system. A drug screening protocol compliant with the new International Council for Harmonisation of Technical Requirements for Pharmaceuticals for Human Use (ICH) guidelines was established and uHeart was employed for testing the effect of 11 compounds acting on single or multiple cardiac ion channels and well-known to elicit QT prolongation or arrhythmic events in clinics. The alterations of uHeart’s electrophysiological parameters such as the beating period, the FP duration, the FP amplitude, and the detection of arrhythmic events prior and after drug administration at incremental doses were effectively analyzed through a custom-developed algorithm. Results demonstrated the ability of uHeart to successfully anticipate clinical outcome and to predict the QT prolongation with a sensitivity of 83.3%, a specificity of 100% and an accuracy of 91.6%. Cardiotoxic concentrations of drugs were notably detected in the range of the clinical highest blood drug concentration (*C*_max_), qualifying uHeart as a fit-to-purpose preclinical tool for cardiotoxicity studies.

Cardiotoxicity and potential pro-arrhythmic effects of drug candidates represent a major drawback in the DDP (Pang *et al.*, 2019). The potential onset of the Torsades de Pointes (TdP), a rare lethal arrhythmia which is linked to an increased clinical heterogeneity modification of the ventricular repolarization, remains of great concern for Pharma and Regulatory Agencies (Fermini and Fossa, 2003). These entities recently defined specifications in the framework of the International Council for Harmonisation of Technical Requirements for Pharmaceuticals for Human Use (ICH) for evaluating QT/QTc prolongation and proarrhythmic potential (E14&S7B) before first-in-human dosing (ICH guideline E14/S7B, 2022; Strauss *et al.*, 2021; Vargas *et al.*, 2021). Preclinical investigation involving *in vitro* and animal experimentations thus play a critical role in determining drug progression or termination in the DDP. In particular, current guidelines (ie, ICHS7B) impose that preclinical *in vitro* studies assess the drug-induced potassium channel blockage (ie, human ether-a-go-go-related gene—hERG assay) before performing telemetry in animals (Garrido *et al.*, 2020; Rock *et al.*, 2009). Despite this approach has been demonstrated to effectively hamper potential torsadogenic drugs to reach the market, its lack of specificity often led to an inappropriate drug attrition and to a premature discontinuation of promising candidates, which while affecting potassium current do not cause TdP in humans (Fermini *et al.*, 2016; Valentin *et al.*, 2022). Several evidences indeed proved that QT prolongation and the connected TdP risk in human can be mitigated by the simultaneous block of inward depolarizing currents (ie, calcium or late sodium), which balances the outward potassium current blockage, such as in the case of verapamil and ranolazine (Johannesen, 2014; Redfern *et al.*, 2003). Animal models have their own set of advantages and limitations. Despite regulatory agencies (eg, EMA, FDA) require cardiotoxicity *in vivo* assessment, nowadays there is an evident need to better integrate *in vivo* outcomes with data derived from more physiological, ideally human-based, *in vitro* models to better translate the findings to clinics (Ewart *et al.*, 2014; Milani-Nejad and Janssen, 2013). Indeed, cardiovascular issues identified in animal models often drive the termination of drug candidates in the preclinical phases without having the confirmation of their real clinical risk. This translate into beneficial treatments being discarded for potential burdens in animals that would not result as burdens in patients (Pang *et al.*, 2019). Moreover animal experimentation is performed in the latest stages of the preclinical phases, reducing the chance for improving molecule designs to actually prevent human relevant drawbacks (Hingorani *et al.*, 2019; Van Norman, 2020). These observations suggest the urgent need for a new and more comprehensive approach for nonclinical/clinical risk assessment, enabling to perform more representative preclinical *in vitro* studies. This may take advantage of engineering cardiac models which better predict the human heart behavior, ultimately improving clinical QTc studies and finally streamlines the drug development (Vargas *et al.*, 2021). In this context, human-induced pluripotent stem cell-derived cardiomyocytes (h-iPSC-CMs) open the path toward superior human-based investigations of drug-related cardiac risks already in the preclinical phases (Denning *et al.*, 2016). Even if the currently available differentiation protocols lead to the generation of cells showing a fetal-*like* rather than an adult-*like* cardiac phenotype, h-iPSC-CMs provide the unprecedented ability to express multiple human-relevant cardiac currents, whose orchestrated operations define the typical cardiac action potential profile (Gowran *et al.*, 2016; Pourrier and Fedida, 2020; Yang *et al.*, 2014). The CiPA initiative, whose outcomes laid fundaments to update the current ICH guidelines E14/S7B (Vargas et al., 2021) by enabling the use of nonclinical approaches to assess proarrhythmic and delayed repolarization risks, builds upon iPSC-CMs to develop new *in vitro* assays for the evaluation of drug-induced cardiac electrophysiologic activity changes in human relevant models (Sager *et al.*, 2014). To date, most of the systems under validation in the framework of CiPA exploit the 2D culture of h-iPSC-CM monolayers on multielectrode arrays (MEA) (Gintant *et al.*, 2020; Millard *et al.*, 2018; Yu *et al.*, 2019). Being embedded in a rigid cell culture substrate, MEAs allow to directly record the cell electrophysiology (ie, FP) in response to compound administration. In particular, the recorded FP signal reflects the depolarization and repolarization phases of the cardiomyocyte (or the group of cardiomyocytes) located around the specific measuring electrode and its changes are correlated to potential cardiac safety liability. MEA-based systems have been deeply characterized and have been demonstrated reliable in blinded and multicentric studies (Blinova *et al.*, 2018; Kanda *et al.*, 2018). However, these assays still rely on the culture of cells in a 2D configuration, which do not resemble the 3D native cardiac microenvironment and thus limit potential cell functionality, partially reached in MEA only by a prolonged time in culture (eg, >10–14 days) (Blinova *et al.*, 2017). Three-dimensional cell cultures indeed have been extensively demonstrated to be more physiologically relevant and predictive than 2D cell cultures (Duval *et al.*, 2017). Taking advantage from 3D spatial cell organization and introduction of relevant native-resembling mechanical stimulation (Carlos-Oliveira *et al.*, 2021), we previously described the development of an iPSC-CMs-based cardiac model (Marsano *et al.*, 2016), named uHeart, able to achieve spontaneous and synchronous beating already after 7 days in culture. Moreover, we integrated a miniaturized electrical system (Visone *et al.*, 2018) to monitor online the entire microtissues’ electrical activity, preliminary proving the possibility to test electrophysiological alterations induced by drugs (Visone *et al.*, 2021).

Here, we describe the results of a pharmacological campaign conducted to qualify uHeart model as a promising preclinical tool for cardiac safety screening. Electrophysiological alterations in response to 11 well-known drugs (among compounds evaluated in the CiPA initiative) administered to uHeart at increasing concentrations were directly measured and analyzed by means of a purposely developed software algorithm. Unique to this study is the direct assessment of the FP alterations and the identification of arrhythmic events within 3D beating cardiac models in response to a broad panel of drugs (belonging to different risks categories and blocking either single or multiple cardiac ion channels) (Colatsky *et al.*, 2016). The goal of the study is the establishment of inclusion criteria, the development of a drug screening protocol, and the setting of reliable analyses methods compliant with current ICH S7B guidelines (ICH guideline E14/S7B, 2022), eventually proving the ability of uHeart to successfully predict concentration dependent drug-induced electrophysiological changes consistent with clinical outcomes.

## Materials and methods

###  

####  

##### uHeart model development

**Figure 1.**
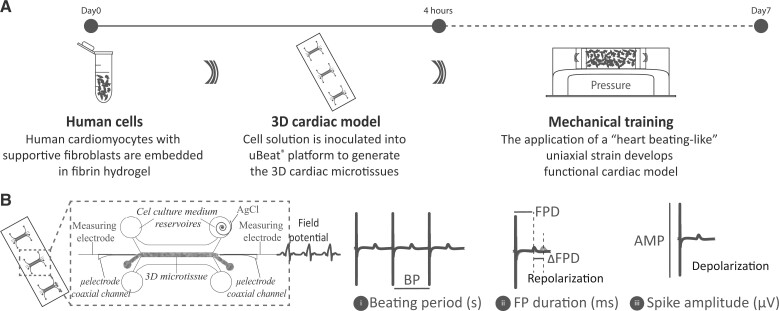
Schematic representation of the experimental setup used to (A) develop the uHeart model and (B) to measure and evaluate the electrophysiological parameters of the cardiac microtissues (BP, FPD, FPD changes and AMP).

H-iPSC-CMs were provided by FUJIFILM Cellular Dynamics Inc. (iCell Cardiomyocytes; donor 11713: healthy Caucasian female, 30–39 years old). Human adult dermal fibroblasts were provided by Lonza Group Ltd. H-iPSC-CMs were thawed according to standard protocols and immediately used. Fibroblasts were expanded at confluency in DMEM (Gibco, 4.5 g l^−1^ glucose) supplemented with 10% v/v FBS, 100 U ml^−1^ penicillin, 100 μg ml^−1^ streptomycin, and 10 mmol HEPES and used at passage 10–11. As previously described (Visone *et al.*, 2021), uHeart models (Figure 1A) were generated by mixing 75% of h-iPSC-CMs with 25% of fibroblasts (Ronaldson-Bouchard *et al.*, 2018). Cells were embedded at a density of 125 *×* 10^6^ cells ml^−1^ in fibrin hydrogel (ie, final concentration of 10 mg ml^−1^ of human fibrinogen and 1.25 U ml^−1^ of human thrombin). Subsequently, 2 μl of cell solution was inoculated into the central channel of each chamber of the uBeat Platform (provided by BiomimX Srl). The hydrogel was cross-linked in the incubator at 37°C for 8 min before manually adding the plating medium (FUJIFILM Cellular Dynamics Inc.) supplemented with 100 U ml^−1^ penicillin, 100 μg ml^−1^ streptomycin, and 2 mg ml^−1^ of 6-Aminocaproic acid (ACA). The mechanical stimulation (ie, 10% uniaxial strain at 1 Hz) (Marsano *et al.*, 2016) was started after 4 h from the seeding. After 24 h, the plating medium was substituted with RPMI (Sigma Aldrich) medium supplemented with 100 U ml^−1^ penicillin, 100 μg ml^−1^ streptomycin, 292 µg ml^−1^ of l-glutamine, 20 µl ml^−1^ of B-27 and 2 mg ml^−1^ of ACA. This medium was used and changed every day for the following 6 days. The amount of ACA in the medium was gradually diminished as previously described (Visone *et al.*, 2021).

##### Field potential measurements

Field potential (FP) measurements (Figure 1B) were performed after 6 or 7 days of culture, when the model expresses a strong spontaneous and synchronous beating. The measuring electrodes (ie, stainless steel microneedles) were precisely positioned in contact with the 2 edges of uHeart by means of the µECG technology (Rasponi *et al.*, 2018). An AgCl ground electrode was inserted in one of the 4 cell culture medium reservoirs. Cell culture medium was changed at least 1 h prior to perform the measurements. To execute the tests, uHeart was maintained in the incubator at 37°C with 5% CO_2_ and the electrodes were connected to an extracellular amplifier (Ext-02b, Npi Electronic GmbH, Germany) with a 1 *×* 10^4^ gain and a bandpass filter between 0.3 Hz and 10 kHz. FPs were acquired with an electronic board (Analog Discovery 2, Digilent, Washington) at a rate of 2000 samples s^−1^. For each uHeart model, 2 FPs were recorded (taken simultaneously at its opposite sides) and the cleaner signal (ie, depolarization and repolarization phases clearly better recognizable) was used to evaluate the following electrophysiological parameters: beating period (BP), FP duration (FPD), FPD corrected with the Fridericia methods (FPD_cF_) (Fridericia, 2003), and spike amplitude (AMP).

##### Compound selection and preparation

Eleven compounds belonging to different drug classes and classified by CiPA at different TdP risk levels (ie, low, intermediate and high) were selected to perform the uHeart qualification study (Table 1). To verify that the assay performed on uHeart is sensitive to drugs affecting hERG, sodium and calcium currents, drugs blocking single cardiac ionic currents, and specifically listed in the ICH S7B (Q&A 2.5) were first chosen as calibration compounds: dofetilide (*I*_kr_), nifedipine (*I*_CaL_), and mexiletine (*I*_Na_). Drugs affecting multiple cardiac ionic currents were selected as additional compounds: sotalol, quinidine, cisapride, terfenadine, ranolazine, verapamil, alfuzosin, and aspirin. Concentrations in the ranges of the *C*_max_ or free effective therapeutic plasma concentration (*f*_ETPC_) and previously tested in MEA systems were selected (Ando *et al.*, 2017; Blinova *et al.*, 2017; Kanda *et al.*, 2018). All compounds were provided by Sigma Aldrich and were prepared in dimethylsulfoxide (DMSO, Sigma-Aldrich) at a concentration 1000 times higher respect to the maximum target concentration used for the drug screening. Serum-free RPMI medium was used to further dilute the compounds to reach the desired concentrations for the test. DMSO, which is the solubilizing agent, was used as vehicle and as negative control (10^−5^%–10^−4^%–10^−3^%–10^−2^%–10^−1^%) as suggested in the ICH S7B best practice guidelines (ICH guideline E14/S7B, 2022).

**Table 1.** List of drugs used in the pharmacological campaign to qualify the uHeart model as detector of functional cardiotoxicity.

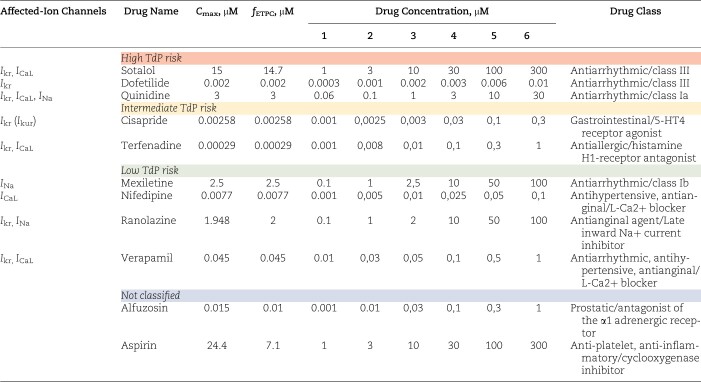

*C*_max_ values from Blinova *et al.* (2017), Kopljar *et al.* (2018), Voelker and Hammer (2012), *f*_ETPC_ from Nguyen *et al.* (2017), Yamazaki *et al.* (2018), and drug classification from Ando *et al.* (2017).

##### Drug screening protocol

Prior to start the assay (Supplementary Figure 1), 2 electrodes were inserted in 2 opposite sides of each microtissue (Visone *et al.*, 2021). The cell culture medium was exchanged with fresh serum-free RPMI medium and after 1 h the baseline FP signal was recorded for at least 3 min. Subsequently, the lowest drug dose was administered to the microtissues and incubated at 37°C for 10–20 min before recording the corresponding FP for at least 3 min. Between each incremental dose, a 5-min washing was performed with serum-free RPMI medium.

##### Semautomatic detection of relevant parameters from µECG signal

The µECG signals were analyzed through a custom-made algorithm developed to identify relevant changes in the beating parameters: BP, FPD, and AMP (defined in Figure 1B), as well as the onset of arrhythmic events following compounds administration. MATLAB (MathWorks) was used to develop the code. In particular, the algorithm encompassed 3 main phases: (1) identification of the depolarization phase for each beat by detecting the peaks in the FP signal using a Pan-Tompkins-like algorithm; (2) preprocessing of the FP signal by an optional zero-phase notch filter (*f* = 50 Hz and its harmonics) and computation of the averaged patterns for depolarization and repolarization, iteratively aligned to the FP signal by cross-correlation; (3) identification of fiducial points first on the average patterns and then on the FP signal, to evaluate the mean and standard deviation of electrophysiological parameters (ie, BP, FPD, AMP). The algorithm also computes the residual of the signal, ie, the difference between the raw recorded FP and the mean of the signal computed through the software by combining the identified average patters, allowing for the evaluation of irregular peaks which may be associated to arrhythmic events. The software was validated by analyzing 21 FP signals, belonging to 3 different cardiac microtissues that were subjected to incremental doses of compounds (ie, nifedipine and dofetilide). Results of BP, FPD, and AMP assessed by the algorithm were compared against manual measurement. BP and FPD were additionally analyzed by applying an initial bandwidth filter to the signal (3rd order Butterworth filter, 0.67–100 Hz, zero phase), to further verify the quality of the estimates obtained. After the validation, the algorithm was used to analyze the FP signal recorded during the pharmacological campaign. Signals not correctly recognized by the software, as verified by visual inspection on the user interface, were manually analyzed.

##### TdP risk scoring

The results obtained from the biological campaign concerning the FPD prolongation and arrhythmic event detection in uHeart were further elaborated to propose a TdP risk score for each compound. The adopted criteria are based on studies that used 2D iPSC-CMs cardiac models (Ando *et al.*, 2017): score from −1 to 3 were assigned and plotted against the ratio between the concentration of drug eliciting a significant response and the *f*_ETPC_. For compounds not evoking responses, the maximum tested concentration respect to the *f*_ETPC_ was used. Three areas were defined on the graph, corresponding to the 3 risk categories: high risk (red, upper and left area) for scores ≥2 at 10-fold ratio, intermediate risk (yellow, middle area) for scores = 1 at ratio between 1 and 100, and for scores >2 at ratio between 10- and 100-fold and low risk (green, bottom and right area) for score = 0 and score ≥1 at ratio higher than 100-fold.

##### Quality control and statistical analyses

A quality control was established to select the signals to be considered for the evaluation of electrophysiological parameters changes in response to increasing concentrations of compounds. In particular, microtissues that in the baseline BP signal (ie, before compound administration) exhibited a coefficient of variation >25% were discarded. This inclusion criteria ensured that the developed cardiac microtissues spontaneously beat in a consistent way and allowed to normalize the obtained FP signals with correction methods based on the beating frequency.

Data were presented as mean ± standard deviation or as mean ± standard error. Statistical analysis was made through Kruskal–Wallis with Dunn’s multiple comparison test (non-normal distributions; ∗*p* < .05; ∗∗*p* < .01; ∗∗∗*p* < .001). Statistical analyses were performed using Graph-Pad Prism.

## Results

###  

#### Semiautomatic analysis of uHeart electrophysiological parameters

**Figure 2.**
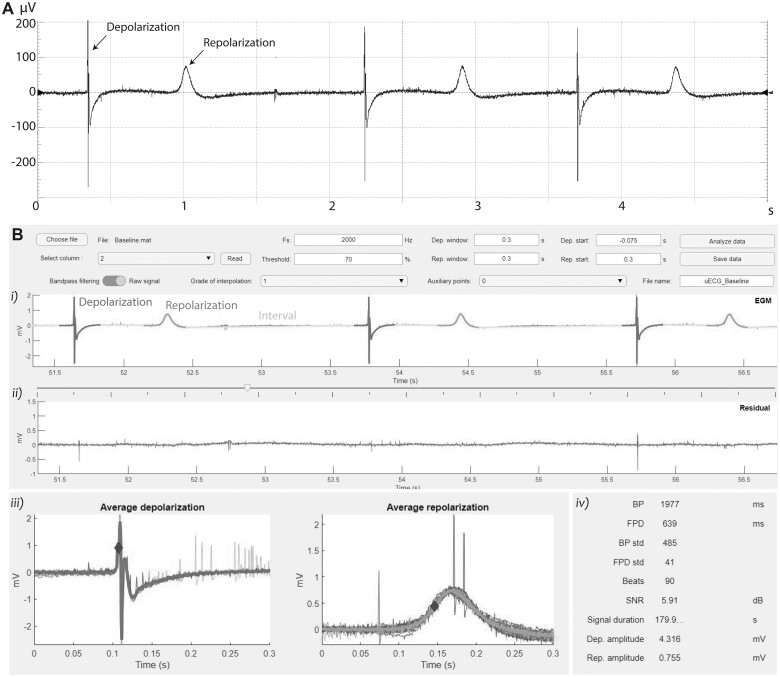
A, Representative FP signal record from uHeart. B, Software developed detect uHeart’s electrophysiological parameters: (1) identification of depolarization spikes, repolarization waves and intervals between the events; (2) residual of the signal after the depolarization and repolarization events are excluded; (3) representation of the averaged depolarizations and repolarizations with reference points indicating the events onset time; (4) means and standard deviations of the computed electrophysiological parameters.

uHeart models started beating already after 3 days in culture, and after 7 days the µECG technology was exploited to acquire the cardiac electrophysiological signals. Figure 2A shows a representative FP recorded, highlighting the typical initial spike corresponding to the depolarization of the cardiac microtissue and the smoother peak related to the repolarization of uHeart. Signals extracted from uHeart were analyzed by means of the software (Figure 2B). By manually setting as input parameters, the sampling frequency of the acquired signal (ie, 2000 Hz), the voltage threshold of the expected spikes (eg, 70% respect to the highest value), the expected depolarization/repolarization windows (ie, usually 0.5 s and 0.7 s, respectively) and the depolarization/repolarization starting (ie, the expected delay of the event from the peak, usually set at −0.075 s and 0.1 s, respectively), the algorithm automatically recognizes the FP patterns. In details, it identifies narrow spikes (ie, depolarization phases), smoother waves (ie, repolarization phase) and the intervals between them. By averaging the captured events, the means and standard deviations of the BP, the FPD, and the AMP, together with the number of recognized beats, the total duration of the signal and the signal to noise ratio (SNR) are computed. Moreover, the residual signal was used to identify potential arrhythmic events, characterized by unexpected and irregular peaks along to electrical signal (Supplementary Figure 2).

The validity of the software was demonstrated by comparing the obtained results with electrophysiological parameters manually evaluated from the same signals (*n* = 21). Supplementary Figures 3A and 3B highlight the accurate overlapping between automatically computed data and manual measures of BP and FPD, both when signals were pre-filtered or analyzed as raw data. Similarly, the AMP computed by the software (Supplementary Figure 3C) resulted accurate respect to manually analyzed data. Overall (Supplementary Figure 3D), the automatically computed filtered signals showed that the software slightly overestimated the BP and FPD respect to the manual elaborated signals with an error of about 6.4% and of 3.8%, respectively. The AMP instead was underestimated, showing a difference of about 12%, when the signals were not filtered.

#### Characterization of uHeart model

**Figure 3.**
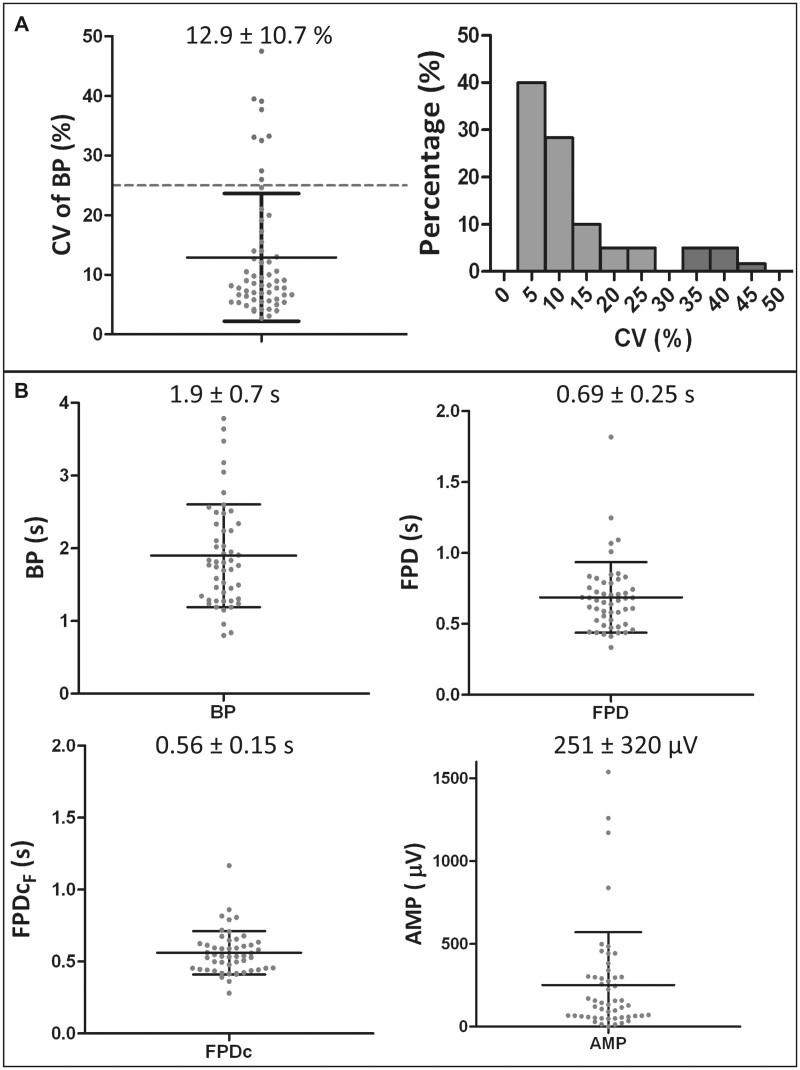
A, Frequency distribution and values of the CV characterizing the 60 generated uHeart. B, Beating period (BP), field potential duration (FPD), FPD corrected with the Fridericia methods (FPDcF) and spike amplitude (AMP) characterizing the uHeart models matching inclusion criteria (*n* = 51). Data are represented as mean ± standard deviation.

In the entire study, 60 cardiac microtissues were generated. Among those, 9 were excluded (Figure 3A, dots above the dotted horizontal line) because they did not match the inclusion criteria (ie, coefficient of variation of the BP in the baseline <25%). As detailed in Figure 3A, more than the 88% of the microtissues showed a CV lower than 25% (groups of bar on the left in the histograms) and, among them, 40% showed a CV lower than 5%, and 28% are characterized by a CV smaller than 10%. The mean CV was around 13%, with a standard deviation of 10.7%. The analyses of the FP baseline performed on the 51 microtissues that matched inclusion criteria allowed to characterize the electrophysiological parameters of the developed cardiac microtissues (Figure 3B) that showed a mean BP of 1.9 ± 0.7 s, a mean FPD of 0.69 ± 0.25 s and FPD_cF_ of 0.56 ± 0.15 s. The FP amplitude was quite variable between microtissues, with a mean value of 251 ± 320 μV and minimum and maximum values of 0.95 µV and 1538 µV, respectively.

#### uHeart responses to calibration compounds

**Figure 4.**
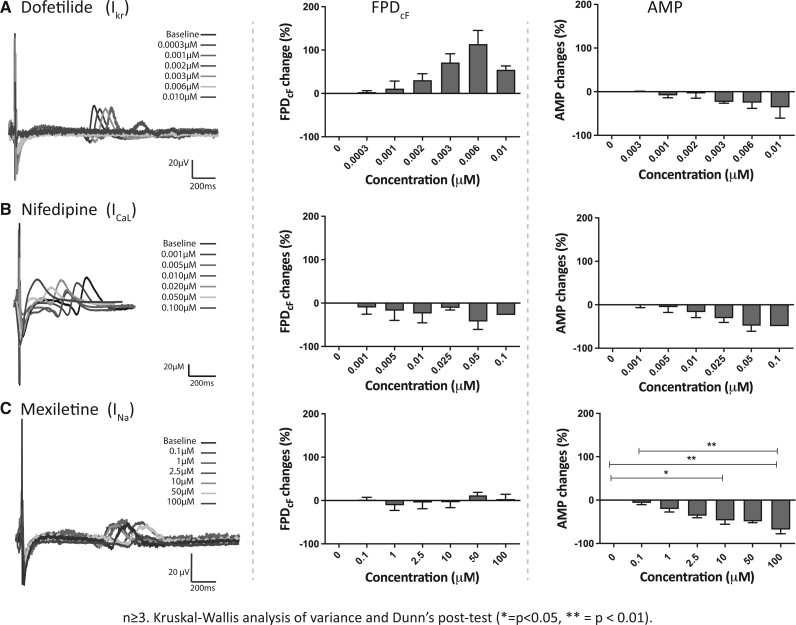
Representative field potentials (FPs) and electrophysiological parameters (ie, BP and FP duration corrected with Fridericia methods) of uHeart microtissues subjected to drugs acting on single cardiac ion channels: (A) dofetilide, (B) nifedipine, and (C) mexiletine. Data are represented as mean ± standard error.

uHeart effectively responded to the selected calibration compounds (Figure 4), as highlighted by the representative waveforms of FP (A–C, left) before (black) and after (colored) exposure to different drug concentrations and as confirmed by the concentration-dependent percentage changes of FPD_cF_ and AMP (A–C, central and right). Dofetilide (*C*_max_ of 2 nM), which selectively blocks the delayed rectifier potassium current (*I*_Kr_), caused a concentration-dependent prolongation of the FPD_cF_ and an incremental decrease in the AMP, especially at high dosage. Of note, 1 over 3 analyzed cardiac microtissues ceased to beat at 0.006 µM. Conversely, nifedipine (*C*_max_ of 7.7 nM), which selectively inhibits L-type calcium current (*I*_CaL_), induced a concentration-dependent shortening of the FPD_cF_. Nifedipine decreased the AMP and caused the cessation of the spontaneous beating at the highest concentration in 1 over 3 samples, effect not reported in literature (Millard *et al.*, 2018). The latest tested compound mexiletine (*C*_max_ of 2.5 µM), acting primarily by blocking the depolarizing sodium inward current (*I*_Na_), did not affect the FPD as highlighted by the absence of dose-related monotonous variations of the FPD_cF_. In contrast, the AMP statistically decreased in a concentration-dependent way, already at medium concentration (ie, 10 µM).

#### uHeart responses to test compounds

**Figure 5.**
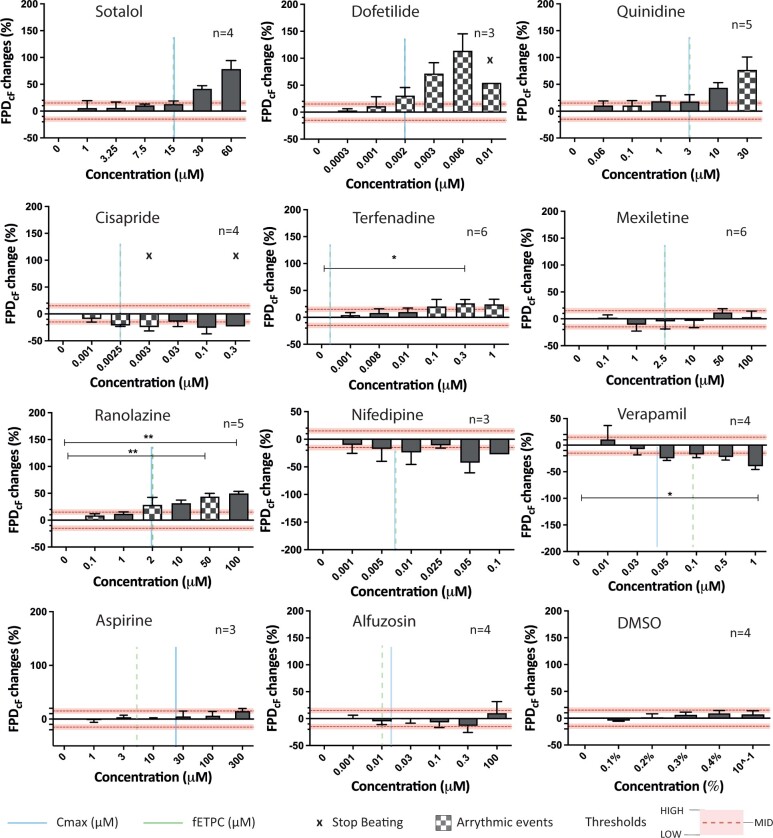
Percentage changes in the FPD corrected with Fridericia formula of uHeart models subjected to drugs acting on single or multiple cardiac ion channels. For each drug, the *C*_max_ (vertical line), the fETPC (vertical dotted line), arrhythmic events (chessboard bars) and the interruption in the spontaneous beating of uHeart (× symbols) are reported in correspondence of the drug concentration. Kruskal-Wallis analysis of variance and Dunn’s post-test (*=*p* < .05, **=*p* < .01.). Data are reported as mean ± standard error.

Figure 5 summarizes the effects of the 11 tested compounds and the vehicle (ie, DMSO) on uHeart’s FPD_cF_, expressed as percental changes respect to the baseline. Histograms with checkerboard pattern highlight concentrations at which onset of arrhythmic events were detected, whereas the “X” symbols indicate the cessation in the spontaneous beating of uHeart. Horizontal dottedlines represent the 15% (ie, MID) arbitrary predefined thresholds, whereas the shadowed area represents a 5% interval, defining an upper limit which corresponds to the HIGH threshold (ie, 20%) and a bottom limit which represents the LOW threshold (ie, 10%), highlighting different level of the FPD_cF_ percental changes respect to the baseline.

As expected, compounds with a high risk of TdP and affecting hERG channel (ie, sotalol, dofetilide, quinidine) effectively prolonged the FPD_cF_ above the defined thresholds and slightly increased the BP (Supplementary Figure 4), especially at high doses. Arrhythmic events were detected in both uHeart administered with dofetilide and quinidine, already at low dosages. Moreover, a high dosage of dofetilide (ie, 0.01 µM) stopped the beating of 1 over 3 tested uHearts. Similarly, cisapride, a drug acting by blocking the *I*_kr_ current and classified as compound with intermediate risk for TdP, caused the termination of the beating in 2 over 4 uHearts at medium (ie, 0.003 µM) or high (ie, 0.3 µM) dosages albeit inducing a slightly decrease in the FPD_cF_. Despite it belongs to the same category, terfenadine statistically prolonged the FPD_cF_ at high dosages (ie, 0.3 µM) without affecting the BP and elicited the onset of arrhythmic beatings already at low concentrations (ie, 0.001 µM). Among drugs with low risk, mexiletine did not show a trend in altering neither the FPD_cF_ nor the BP, whereas ranolazine statistically enhanced the repolarization time starting from concentrations near the *C*_max_ (ie, 2 µM), rose the BP and generated arrhythmia. On the contrary, nifedipine and verapamil, both acting on *I*_Ca_, showed a tendency to shorten the FPD_cF_ passing the HIGH threshold already at medium dosages. Among the nonclassified compounds, aspirine showed an increment of the FPD_cF_ just above the LOW threshold only at the highest concentration tested (ie, 300 µM). Alfuzosin did not showed a peculiar trend, it slightly shorten the FPD_cF_ at medium-high concentration (ie, 0.3 µM) and prolonged almost of 10% the FPD_cF_ at high concentration (ie,100 µM). The DMSO, used as vehicle, did not caused an evident alteration neither of the FPD_cF_ nor of the BP, even at very high concentrations. The detailed FPD_cF_ changes for each uHeart is shown in Supplementary Figure 5 and representative arrhythmic events (one for each drugs eliciting them) are displayed in Supplementary Figure 6. Except for cisapride, AMP was slightly decreased especially at high dosages in all the conditions tested, with a statistically significant difference only in uHearts administered with mexiletine (Supplementary Figure 7).

#### Comparison between uHeart categorization and FDA labels

The effects of all compounds tested on uHeart are summarized in Table 2. Drugs were classified based on their CiPA risk and by assessing the indications reported on the FDA label, as previously detailed in the paper of Blinova and colleagues (Blinova *et al.*, 2017). The table shows the concentration causing an FPD_cF_ alteration (ie, ↑prolongation, ↓shortening, ↔no effect) greater than a fixed threshold (ie, LOW, MID, HIGH) and its fold increase respect to the *C*_max_, the dose eliciting arrhythmic events (ie, the lowest) detected concentration and the percentage of uHearts affected) and inducing the beating cessation (ie, percentage of uHearts). Correct compound classification for each considered threshold is highlighted with a green square, whereas wrong classification with a red one.

**Table 2.** Classification of the tested compounds based on the risk to cause QT prolongation and TdP as referred on the FDA label, with corresponding CiPA risk categories (H = high; I = intermediate; L = low).

		uHeart							
Risk CiPa	Drug	[%FPD_cF_] >_LOW_ (µM)	[%FPD_cF_] >_MID_ (µM)	[%FPD_cF_]>_HIGH_ (µM)	Arrhythmic events	Stop	[%FPD_cF_>_LOW_]/*C*_max_	[%FPD_cF_>_MID_]/*C*_max_	[%FPD_cF_>_HIGH_]/*C*_max_
QT ↑ and TdP on FDA label									
H	Sotalol	↑7.5 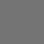	↑30 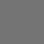	↑ 30 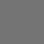	N	N	0.5	2.0	2.0
H	Dofetilide	↑0.001 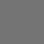	↑ 0.002 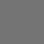	↑ 0.002 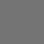	Y (0.001)—66%	Y (33%)	0.5	1.0	1.0
H	Quinidine	↑ 0.06 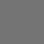	↑ 1 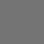	↑ 10 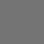	Y (0.1)—50%	N	0.02	3.33	33.3
I	Cisapride	↓ 0.0025 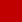	↓ 0.0025 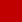	↓ 0.0025 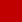	Y (0.001)—50%	Y (25%)	1.0	1.0	1.0
I	Terfenadine	↑ 0.1 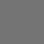	↑ 0.1 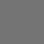	↑ 0.1 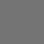	Y (0.001)—60%	n	349.7	349.7	349.7
QT ↑, but NO TdP on FDA label									
L	Ranolazine	↑ 1 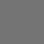	↑ 2 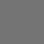	↑ 2 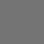	Y (2)—20%	N	0.5	5.1	5.1
NO QT ↑ and NO TdP on FDA label									
L	Mexiletine	↑ 50 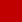	↔ 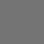	↔ 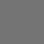	N	N	20	–	–
L	Nifedipine	↓ 0.001 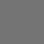	↓ 0.005 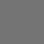	↓ 0.01 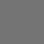	N	N	0.13	0.65	1.3
L	Verapamil	↓ 0.010 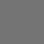	↓ 0.05 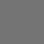	↓ 0.05 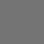	N	N	0.2	1.1	1.1
n.a.	Alfuzosin	↓ 0.3 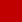	↔ 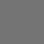	↔ 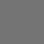	N	N	3	–	–
n.a.	Aspirin	↔ 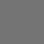	↔ 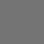	↔ 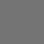	N	–	–	–	
n.a.	DMSO	↔ 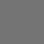	↔ 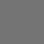	↔ 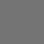	N	–	–	–	

Drug concentration inducing: a field potential prolongation (↑), shortening (↓), or no effect (↔) in uHeart by 10% (FPD_cF_≥_LOW_), 15% (FPD_cF_ ≥ _MID_), or 20% (FPD_cF_≥_HIGH_); correct (green square) and incorrect (red squared) classification of the compounds; the onset of arrhythmic evets; the spontaneous beating cessation. Ratio between effective concentration in uHeart and drug *C*_max_ for the threshold values (ie, LOW, MID, and HIGH).

Among the 6 compounds known to enhance the QT in patients, 5 (ie, sotalol, dofetilide, quinidine, terfenadine, and ranolazine) prolonged the FPD_cF_ in uHeart and one (ie, cisapride) shortened the repolarization time at all considered thresholds. As expected, the concentration eliciting a significant effect (ie, the percental change in FPD_cF_ exceeding one of the fixed thresholds) increased as the chosen threshold rose. As exceptions, cisapride and terfenadine respectively decreased and increased the FPD_cF_ beyond all the defined thresholds at the same concentrations.

Among the drugs not recognized to cause a QT prolongation in patients, nifedipine and verapamil were confirmed to shorten the FPD_cF_ in uHeart, whereas aspirin and DMSO did not change the FPD_cF_. Conversely, mexiletine, and alfuzosin respectively caused the prolongation or shortening of the FPD_cF_ when considering the 10% threshold, but did not affect the repolarization duration if considering the MID and HIGH thresholds.

Arrhythmic events were successfully detected in 66%, 50%, 50%, and 60% of uHearts respectively tested with dofetilide, quinidine, cisapride, and terfenadine, in accordance with the FDA label reporting Risk of TdP. In contrast, sotalol did not elicit any arrhythmia despite its known risk for TdP, whereas ranolazine and alfuzosin caused arrhythmic beating in uHeart, although the TdP is not reported in the label as side effect. Moreover, the 33% and 25% of uHearts administered respectively with dofetilide and cisapride stopped beating at 3-fold and 1.2-fold *C*_max_.

Among the selected thresholds, LOW did not allowed the correct classification of mexiletine as drug not causing a QT prolongation, whereas both MID and HIGH thresholds resulted suitable to correctly categorized all the tested compounds. In details, the MID threshold (respect to the HIGH) guaranteed to correctly recognize the effect of the drugs at a concentration closer to the *C*_max_ in the case of quinidine and nifedipine, resulting a more appropriate cut-off to analyse data from uHeart. In particular, in the case of quinidine, by choosing the HIGH threshold, the ratio between the concentration affecting the FPD and the *C*_max_ resulted 10 times higher compared with the one calculated by selecting the MID one (ie, [%FPD_cF_ > _MID_]/*C*_max_ = 3.33; [%FPD_cF_ > _HIGH_]/*C*_max_ = 33.3). For nifedipine the ratio was almost doubled (ie, [%FPD_cF_ > _MID_]/*C*_max_ = 0.65; ratio between [%FPD_cF_] > _HIGH_]/*C*_max_ = 1.3).

#### uHeart sensitivity, specificity, and accuracy

**Figure 6.**
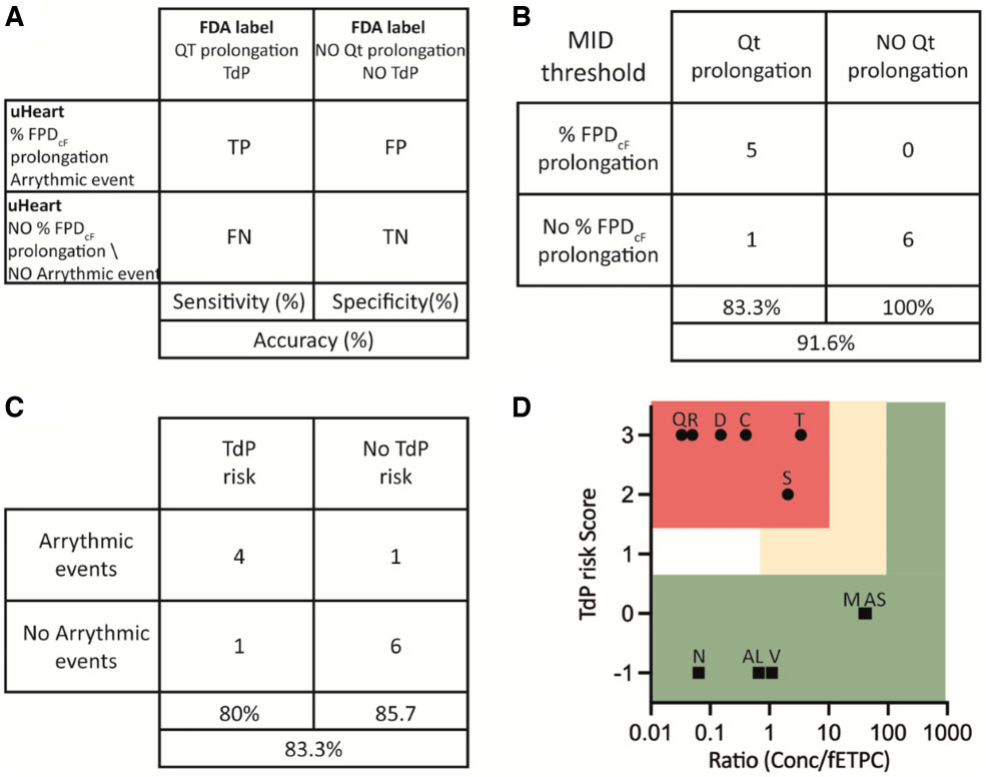
A, Schematic representation of the concordance analyses and sensitivity, specificity, and accuracy parameters performed by comparing the results obtained in uHeart with the FDA drug labels. B, uHeart models is considered to show a field potential prolongation when the percentage changes in the FPDcF is higher than the MID threshold. C, uHeart sensitivity, specificity and accuracy for detecting arrhythmic events, correlating with the risk of TdP onset. D, TdP Risk categorization depicted through two-dimensional map indicating high risk drugs (●) in the top left area, intermediate risk drugs (▲) in the empty middle area and low risk drugs (■) in the bottom and right area. The area not enclosed in rectangle indicates insufficient margins to categorize the compound (risk score 1, margin ≤ 1). Letters on the symbol indicate the first letter of each tested dug (for Alfuzosine-AL and Aspirine-AS).

To qualify the uHeart model as detector of functional cardiotoxicity, true positive (TP), true negative (TN), false positive (FP), and false negative (FN) drugs were defined by correlating the alteration in the uHeart signals with the FDA label indication concerning the QT prolongation and the risk to generate the TdP (Figure 6A). By considering the previously defined optimal threshold of 15%, sotalol, dofetilide, quinidine, terfenadine, and ranolazine were detected as TP drugs, mexiletine, nifedipine, verapamil, alfuzosin, aspirine, and DMSO were revealed as TN, whereas cisapride was identified as FN. These results demonstrated that uHeart has a Sensitivity of 83.3%, a Specificity of 100% and an Accuracy of the 91.6% for detecting the drug-induced QT prolongation (Figure 6B). Similarly, by analyzing the arrhythmic events, uHeart allowed to correctly predict the positive effect of dofetilide, quinidine, cisapride, and terfenadine (ie, TP), the negative behavior of mexiletine, verapamil, nifedipine, aspirine, and DMSO (ie, TN) and mismatched the action of ranolazine and alfuzosin (ie, FP) and sotalol (ie, FN). These results characterized uHeart as an assay with a Sensitivity of 80%, a Specificity of 85.7% and an Accuracy of the 83.3% for predicting the TdP risk (Figure 6C). Supplementary Figures 8A and 8B show the uHeart Sensitivity, Specificity and Accuracy parameters for detecting FPD changes in case the threshold is fixed LOW or HIGH. Moreover, Supplementary Figure 8C provides the total Receiver Operating Characteristic (ROC) curve depicting the variation of these parameters at the different described thresholds as compared with a very low (ie, 5%-INF) and a very high (ie50%-SUP) limits. Based on these results, a categorization of the compounds for torsadogenic potential was also proposed, as depicted in the 2 dimensional maps of Figure 6D. For each drug, a score was assigned based on the %FPD_cF_ respect to the MID and HIGH thresholds and arrhythmic event detection: score −1 for molecules showing a %FPD_cF_ < MID, score 0 for drugs not affecting the FPD_cF_ above the MID threshold, score 1 for compounds showing MID < %FPD_cF_ < HIGH, score 2 for drugs having %FPD_cF_ > HIGH and score 3 for products eliciting arrhythmia. The upper margin defining the high and intermediate risk categories were set at 10- and 100-fold of the ratio between the effective concentration and the *f*_ETPC_, respectively. Six compounds were classified as high risk and the 5 remaining drugs as low risk. Compounds with reported TdP risks (sotalol, dofetilide, quinidine, cisapride, and terfenadine) were located in the high risk area, whereas only 5 (mexiletine, nifedipine, verapamil, alfuzosin, and aspirin) of the 6 drugs without reports of TdP were located in the low-risk area. Ranolazine were classified in the high-risk area, despite it is not reported to elicit arrhythmic events in clinic.

## Discussion

Here, we described the results of a pharmacological campaign conducted to qualify a 3D beating human heart on chip model (namely uHeart) as a preclinical candidate tool for cardiac safety screening. Eleven drugs were selected among compounds previously examined in the CiPA initiative and administered to uHeart at increasing concentrations. Specifically, tested drugs elicit QT prolongation or arrhythmic events in clinics, as defined by FDA label and block single or multiple cardiac ion channels (Colatsky *et al.*, 2016). Upon establishment of inclusion criteria and a testing protocol for drug screening compliant with ICH S7B guidelines (ICH guideline E14/S7B, 2022), the direct assessment of FP alterations and arrhythmic events was conducted by means of an *ad hoc* designed algorithm that elaborated the microtissue electrical activity. The pharmacological campaign allowed for a precise calibration of the system, defining the best threshold (ie, MID) which provides the correct detection of the effects of the drugs, proving uHeart able to successfully predict concentration dependent drug-induced electrophysiological changes consistent with clinical outcomes.

The cardiac microtissues generated with uHeart resulted functional in terms of spontaneous and synchronous beating already after 5/7 days in culture, whereas prolonged culture times (eg, higher than 9 days) resulted in microtissues with reduced or lost spontaneously beating capabilities. An inclusion criterion, indicating a stable electrical activity of uHeart and based on a low BP coefficient of variations, was applied as appropriate experimental control and was met in about 85% of the samples. Despite higher respect to the selected threshold for 2D iCell-based systems (ie, CV ≤ 5% (Blinova *et al.*, 2017) this limit was demonstrated adequate to define 2 subgroups in our uHeart sample population and to ensure that included samples exhibit the expected BP and FPD. These results proved that uHeart cardiac microtissues can be efficiently achieved in less than one week exhibiting a consistent electrophysiological behavior, reducing the experimental time with respect to traditional 2D preclinical models (ie, 12 days) and in particular to *in vivo* animal studies (ie, 2–4 weeks for animal preparation and *in vivo* assessment) (Pang *et al.*, 2019). Moreover, the overall costs to generate the model, mainly impacted by the purchase of cells, remain in the range of standard 2D mid-throughput systems, because the number of cells required to generate the uHeart is comparable with those used to obtain an interconnected monolayer in a well of a standard 48-multiwell plate.

Similarly to previously reported data (Blinova *et al.*, 2018; Kanda *et al.*, 2018), the uHeart electrophysiological parameters did not completely match those proper of the adult human myocardium (Trautwein *et al.*, 1962), probably reflecting the well-known different level of maturation of the starting iCell cardiomyocytes (Kanda *et al.*, 2018). However, thanks to the beating synchronicity of the developed microtissues, the recorded FP signals resulted clean and the typical depolarization and repolarization spikes were easily detected. This allowed the development of the algorithm, which reduced by 75% the post processing data analyses time respect to the manual method (Visone *et al.*, 2021). Of note, although in the canonical MEA 2D system the “gold standard” FP needs to be selected among all the different FPs recorded due to the asynchronous beating of the cells (Millard *et al.*, 2017), uHeart returns a global FP, fully characteristic of the entire developed microtissue. Given this unique advantage of the here described model, the algorithm not only effectively calculates the electrophysiological parameters and their variations upon drug treatments, but also allows for identification of local FP irregularities (ie, features not belonging to the main signals), eventually recognized as arrhythmic events. The automatic detection of parameters such as BP and FPD resulted accurate, especially at lower drug concentrations. At higher dosages, an enhanced variability was instead found, probably reflecting a loss of cardiac microtissues integrity due to incremental compound toxicity, which also resulted in FP with lower signal to noise ratio. This may be corrected in the future by reducing the number of incremental doses tested on a single sample or introducing single dose assays. The amplitude of FP resulted quite variable among the microtissues, but the depolarization spikes remained always recognizable, especially in unfiltered signals. This facilitated the detection of arrhythmic events, that were successfully recognized and could be exploited in the future for the automatic classification of the type of electrical activity abnormalities detected.

The pharmacological campaign conducted with 11 drugs belonging to different classes of risk to determine the TdP, qualified uHeart as a valuable tool to effectively predict the functional cardiotoxicity of compounds and demonstrated the compliance of the assay with the current ICH S7B guidelines (ie, best practice considerations for *in vitro* studies—Q&A 2.2–2.5), validating the test as a promising nonclinical strategy for improving cardiac safety (ICH guideline E14/S7B, 2022). Because the drug screening analyses was performed on spontaneously beating microtissues, the FPD changes were corrected for the BP by using the Fridericia’s method, that was already demonstrated suitable for interpreting results in uHeart (Visone *et al.*, 2021) and allowed a better comparison of data with previously reported studies (Ando *et al.*, 2017; Blinova *et al.*, 2017, 2018; Millard *et al.*, 2018; Yamamoto *et al.*, 2016). As expected, hERG channel inhibitors such as sotalol, dofetilide, quinidine, and ranolazine caused a dose-related FPD_cF_ prolongation independently from their classifications, confirming findings reported in previous studies (Blinova *et al.*, 2017; Kanda *et al.*, 2018; Millard *et al.*, 2018). Moreover, the same compounds were also found to elicit arrhythmic events at different dosages without causing beating cessation, except for dofetilide that interrupted the cell activity at 3-fold the *C*_max_. These confirmed both previously reported data with MEA (Ando *et al.*, 2017; Blinova *et al.*, 2017) and matched the indications of FDA drug labels, reporting a known risk to cause QT prolongation and TdP in clinics. Among these drugs, only sotalol was not found to provoke arrhythmic events, in contrast to previous reports and clinical outcome (Ando *et al.*, 2017). Its action of slowing down the heart beat (Funck-Brentano *et al.*, 1990), was instead recapitulated in uHeart, which showed a drug dose dependent increase of the BP. Of note, terfenadine, an antihistamine withdrawn from the US market for eliciting cardiac arrhythmia triggered by QT interval prolongation (Darpö, 2007), was successfully recognized in uHeart as compound eliciting both a prolongation of the FPD_cF_ and arrhythmic events, conversely to MEA systems which classified the drug as false negative (Ando *et al.*, 2017; Blinova *et al.*, 2017). Ranolazine was reported to increase the BP, accordingly to its mild β-adrenergic receptor blocking effect in human (Kaplan *et al.*, 2022), and to elicit arrhythmia, as previously found using iCell in 2D (Blinova *et al.*, 2017; Kanda *et al.*, 2018; Millard *et al.*, 2018), but in contrast with its classification as drug with low risk for TdP and clinical trials evidences, describing the use of the drug to manage atrial and ventricular arrhythmias in patients (Saad *et al.*, 2016). In contrast, cisapride did not revealed a FPD_cF_ prolongation as expected from the FDA label and as reported from MEA studies. However, it remarkably caused arrhythmic beats and interrupted the spontaneous beating of microtissues, coherently with the effects reported on the FDA label, which were not detected in MEA studies (Blinova *et al.*, 2017). Negative compounds, such as nifedipine and verapamil, well recognized to block *I*_CaL_, shorten the FPD_cF_ in uHeart, coherently with previous experimental reports (Clements and Thomas, 2014; Johannesen, 2014; Millard *et al.*, 2018; Navarrete *et al.*, 2013). Moreover, mexiletine caused a dose-related statistically significant reduction of the FP amplitude without majorly affecting the FPD_cF_ or the BP, in line with its sodium channel blocker function. These results are in line with effects reported on FDA label and in a CiPA study (Blinova *et al.*, 2017), but in contrast with other previous reports applying iCell (Ando *et al.*, 2017; Millard *et al.*, 2018), which failed to correctly detect the compound action. Alfuzosin, an alpha-blocker, was not reported to cause an FPD_cF_ in uHeart, but evidenced the ability to elicit arrhythmia. In clinical studies, the use of this molecule is still controversial, because it has been listed as a drug that just potentially causes QT interval prolongation and/or TdP, despite substantial evidences on this effect are still lacking (Lepor *et al.*, 2008). Moreover, the uHeart not only predicted the expected outcome, but the effective drug concentrations eliciting the response was close to (ie, < 10-fold increase) the clinical *C*_max_ for all the tested compounds, except for terfenadine. Confirming previously reported data (Ando *et al.*, 2017), terfenadine showed to affect the electrical activity of the cells at concentration 350 times higher than the *C*_max_ or the *f*_ETPC_ (ie, 0.29 nM). In our system this may be explained by the fact that the compound is known to be absorbed by PDMS, despite the phenomenon is reported for long incubation (ie, reduction of about 20% after 7 days) (Mair *et al.*, 2022) and not characterized in short-term experiment (ie, some hours). Another reason can be related to the drug metabolism, that is present *in vivo*, but not recapitulated *in vitro* (Leishman, 2020). Indeed we here compared the concentration found effective in uHeart (ie, 100 µM) with the clinical values of *C*_max_ and *f*_ETPC_ of patients, whose metabolism play a pivotal role in transforming terfenadine into Fexofenadine. Conversely, by comparing *f*_ETPC_ obtained in patients in the presence of metabolic inhibition (ie, cytochrome P_450_ inhibitor), reported to be about 4 nM, the concentration results only 25 times lower compared with the effective concentration found in our uHeart model. Moreover, the results are also in line with clinical reports, where an increased *f*_ETPC_ of terfenadine in the presence of metabolic inhibition was associated with delayed cardiac repolarization (Redfern *et al.*, 2003).

Despite being tested on a limited number of molecules respect to CiPA studies (Blinova *et al.*, 2017) (ie, 11 compounds vs 28), overall uHeart showed the same specificity (ie, 100%) and a slightly superior sensitivity (ie, 83% vs 80%) and accuracy (ie, 91.6% vs 86%) respect to 2D iCell cultured on MEA in detecting FPD changes induced by compounds. The strongest contribution however was related to the detection of arrhythmic events, where uHeart demonstrated a sensitivity of 80%, a specificity of 75% and an accuracy of 78%, respect to 2D MEA-based systems (ie, 50%, 66% and 57% respectively).

###  

#### Study limitations

The model here developed is based on cardiomyocytes derived from iPSCs. Despite promising in the field of tissue engineering and personalized medicine, the maturation level of these cells is still far from adult cardiomyocytes (Gintant *et al.*, 2020). Further investigation and optimization of the cell culture protocols are thus required to achieve the desired phenotypic resemblance to the adult human myocardium. The implementation of a 3D model combined with a relevant mechanical training represents a step towards a better mimicking of the *in vivo* heart tissue conditions with respect to simplistic 2D models, allowing for developing microtissues beating as a syncytium and showing more robust prediction of clinical outcome than traditional preclinical models. To further improve the similarity to the native tissue, the adoption of more relevant cell types could be also pursued. By substituting the dermal fibroblasts used in coculture with iPSC-CMs (originally exploited to develop relevant 3D cardiac models by (Ronaldson-Bouchard *et al.*, 2018) with cardiac fibroblasts, a better electrical coupling with cardiomyocytes (Giacomelli *et al.*, 2020) and improved electrophysiological properties of uHeart would probably be achieved. Prolonged culture time has also been proposed as a strategy to strength the maturation level of *in vitro* model (Lewandowski *et al.*, 2018) and the impact of such aspect in the development of uHeart (eg, boosting electrophysiological parameters and lowering the BP coefficient of variation) needs further investigation. Moreover, the occurring changes in the spontaneous beating rate of the tested microtissues registered during drug administration required the use of empirical formula (eg, Fridericia’s, Bazett’s, others.) to derive the net effect of the compounds on the cardiac repolarization. However, these corrections rely on approaches used with *in vivo* data, which are blindly translated to interpret hiPSC-derived cardiomyocyte results assuming that there’s a univocal dependency of FPD and BP. Finer strategies could be further investigated to obtain a more comprehensive understanding of drug effects, such as the one presented in Rast *et al.* (2016) where unique interdependency between the FPD and BP was characterized for each analyzed cardiac model and a square polynomial curve was proposed to better explain the correlation between these 2 parameters. The administration of sequential and incremental doses of drugs to the same microtissue, allowing on one side for the generation of more data points from a single sample, may on the other side increase the risk to introduce confounding effects. The time required to run an experiment is indeed longer and the microtissues may become stressed from the continuous administration of solutes, especially in comparison with single dose administration protocols. This may explain why at increasing doses of a compound, the amplitude of the recorded FP signal from uHeart is gradually reduced, even when the drug does not directly affect the sodium current. Moreover, this protocol may also contribute to incrementally disrupt the physical connection between the cells in uHeart, enhancing the probability to detect arrhythmic events (ie, group of cells may detach from the syncytium and start beating at their own rhythm). This may explain why Ranolazine has been identified by uHeart as a false positive arrhythmogenic compounds and why cisapride, despite not detected as compound affecting repolarization, could still be categorized as high Tdp risk drug as it triggered arrhythmic events. Finally, to be more compliant with the currently ICH S7B guidelines, future satellite experiments are also envisioned to characterize drug exposure profile during the test, by implementing media sampling and drug concentration analyses from uHeart.

## Conclusions

Here we qualified uHeart, a functional 3D model of the human cardiac tissues, as a preclinical tool for detecting functional cardiotoxicity *in vitro*, by predicting the effects of 11 compounds listed in the CiPA studies. The model allowed to predict with high sensitivity, specificity and accuracy how the drugs affected the cardiac BP, FPD, AMP, and the stability of the electrical activity (ie, arrhythmias). The software that we implemented allowed to validate the model and will be further developed to achieve the automatic detection and classifications of the arrhythmic events. Thanks to the fully human setup, uHeart showed great potentiality to supplement and advance limited data currently provided by *I*_kr_ assay and telemetric studies on animal, better matching clinical outcomes in cardiac safety. By taking advantage from the high versatility of the platform and from its compatibility with different readouts, future investigations will be aimed at expanding the use of uHeart in different contexts of use, such as detecting drug structural cardiotoxicity and inotropic effects.

## Supplementary data

Supplementary data are available at *Toxicological Sciences online.*

## Acknowledgments

The silicon wafer micropatterning was performed at PoliFAB, the micro- and nanofabrication facility of Politecnico di Milano. The production of PDMS devices was performed at the Mimic Lab of Politecnico di Milano. Michele Ganelli contributed to the development of a preliminary version of the algorithm used for the uHeart’s electrophysiological signals analyses at the BiSP Lab of Università degli Studi di Milano.

## Funding

This project has received funding from the European Union’s Horizon 2020 research and innovation programme under the Marie Skłodowska-Curie grant agreement No 860715.

## Declaration of conflicting interests

R.V., A.R., M.R., and P.O. share equities in BiomimX Srl.

## Supplementary Material

kfac108_Supplementary_Data
