# Supplementary material for: Predicting human cardiac QT alterations and pro-arrhythmic effects of compounds with a 3D beating heart-on-chip platform
Source: Toxicol Sci. 2022 Oct 13;191(1):47–60. doi: 10.1093/toxsci/kfac108 (PMC9887672; doi:10.1093/toxsci/kfac108)
Supplement: kfac108_Supplementary_Data [file kfac108_supplementary_data.docx]

SUPPLEMENTARY FIGURES

Predicting human cardiac QT alterations and pro-arrhythmic effects of compounds with a 3D beating heart-on-chip platform

Roberta Visone^1,2,^*, Ferran Lozano-Juan^1,2,^*, Simona Marzorati^3^, Massimo Walter Rivolta^4^, Enrico Pesenti^3^, Alberto Redaelli^1^, Roberto Sassi^4^, Marco Rasponi^1^ and Paola Occhetta^1,2^

1 Department of Electronics, Information and Bioengineering, Politecnico di Milano, Milan, Italy

2 BiomimX Srl, Milan, Italy

3 Accelera Srl, Milan, Italy

4 Department of Computer Science, Università degli Studi di Milano, Milan, Italy

* These authors contributed equally to this work

Supplementary Figure 1


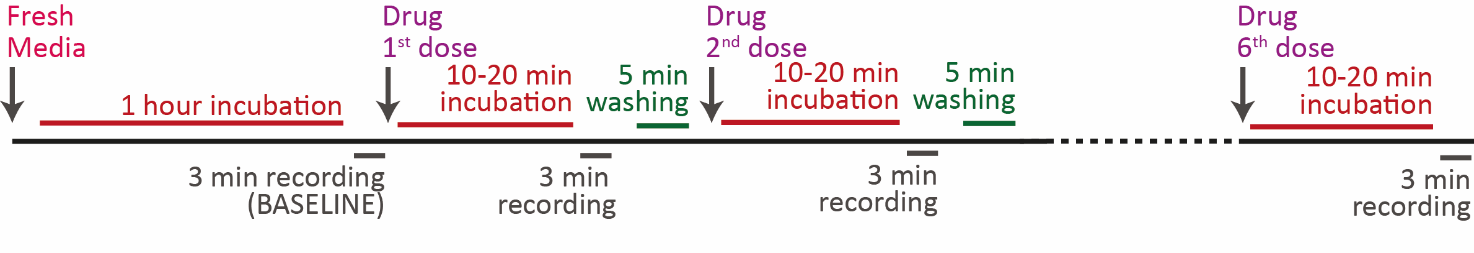


**Suppl Fig. 1**: Schematic representation of the experimental protocol used to perform cardiotoxicity drug screening in uHeart.

Supplementary Figure 2


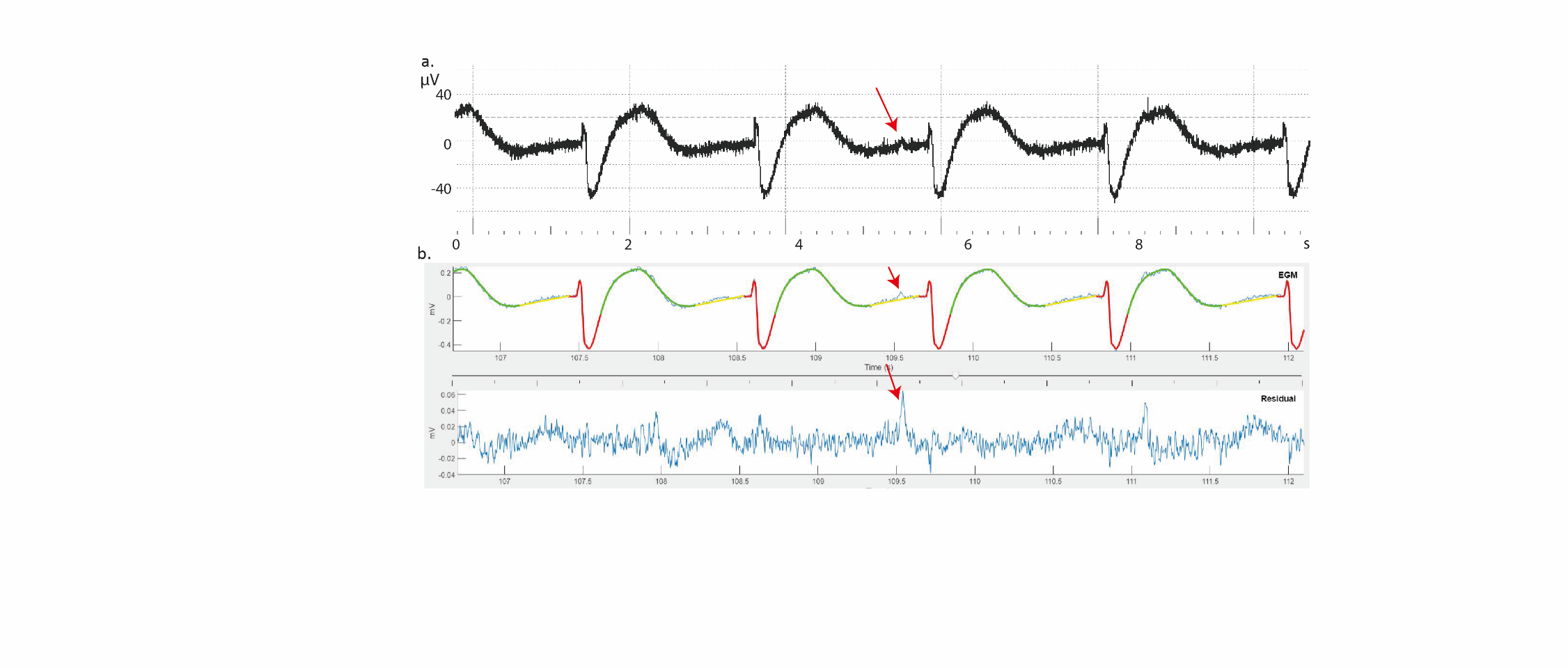


**Suppl Fig. 2**: Example of arrhythmic events (red arrow) detected in a) the acquired FP signal and b) in the software elaborated signal as well as in the residual.

Supplementary Figure 3


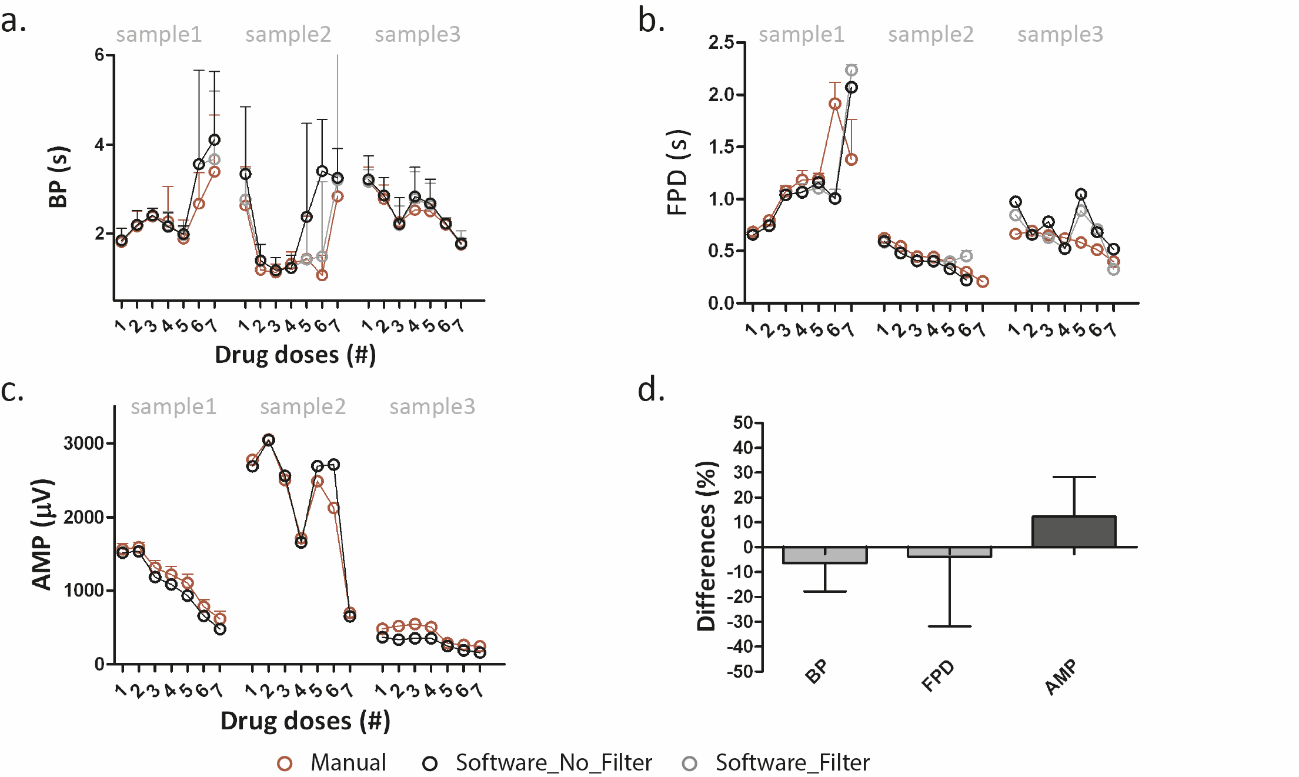


**Suppl Fig. 3**: Comparison of electrophysiological parameters computed manually or with the software: a-c) BP, FPD and AMPL and d) mean percentage differences. Data variation, which occurred most at high drug dosages, not influenced the electrophysiological parameter trend that remained coherent with manual detection.

Supplementary Figure 4

##
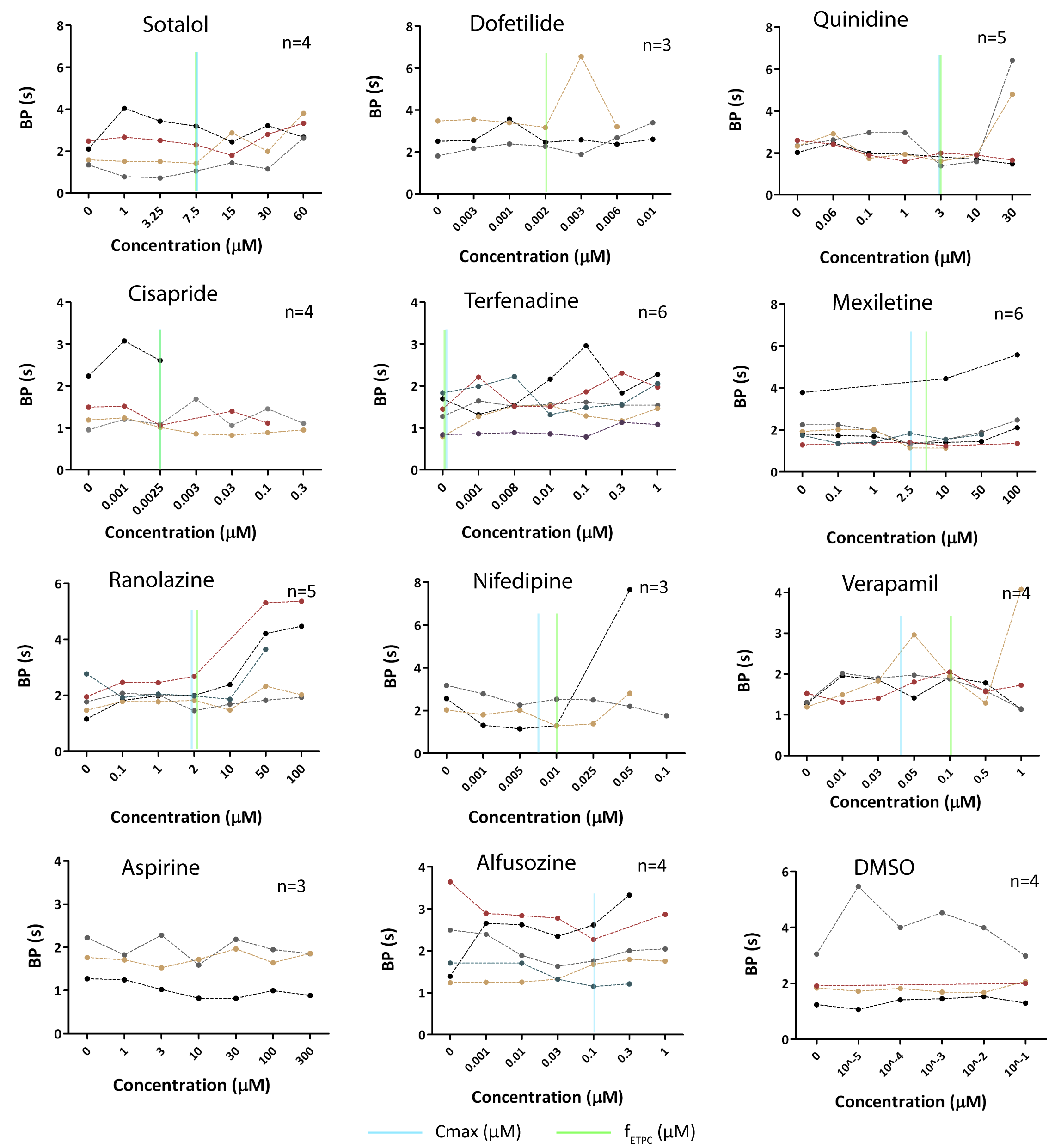

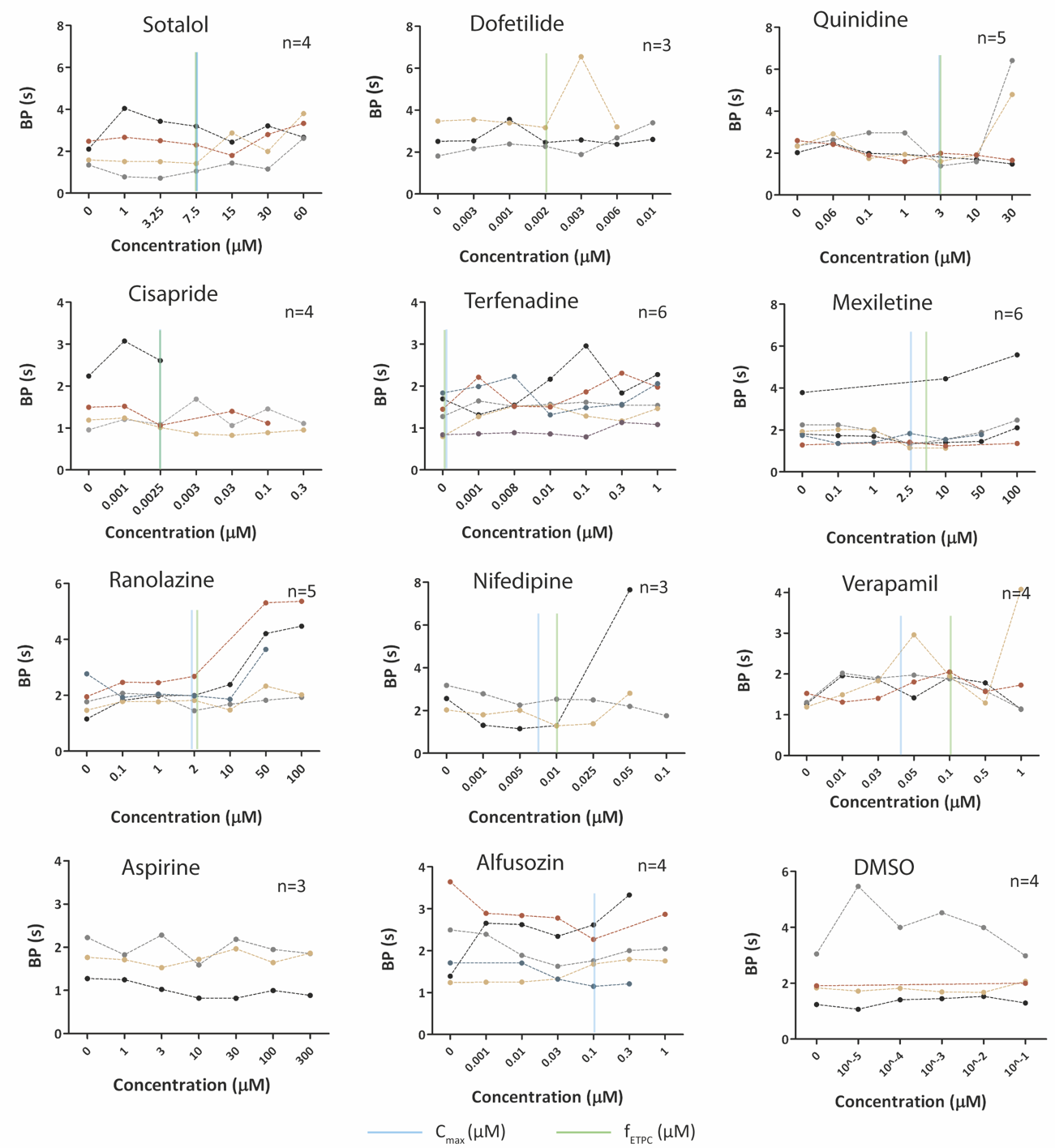


**Suppl Fig. 4**: Beating period of each uHeart model subjected to different concentrations of drugs acting on single or multiple cardiac ion channels. For each drug, the C_max_ (vertical blue line) and the f_ETPC_ (vertical green line) are reported.

Supplementary Figure 5


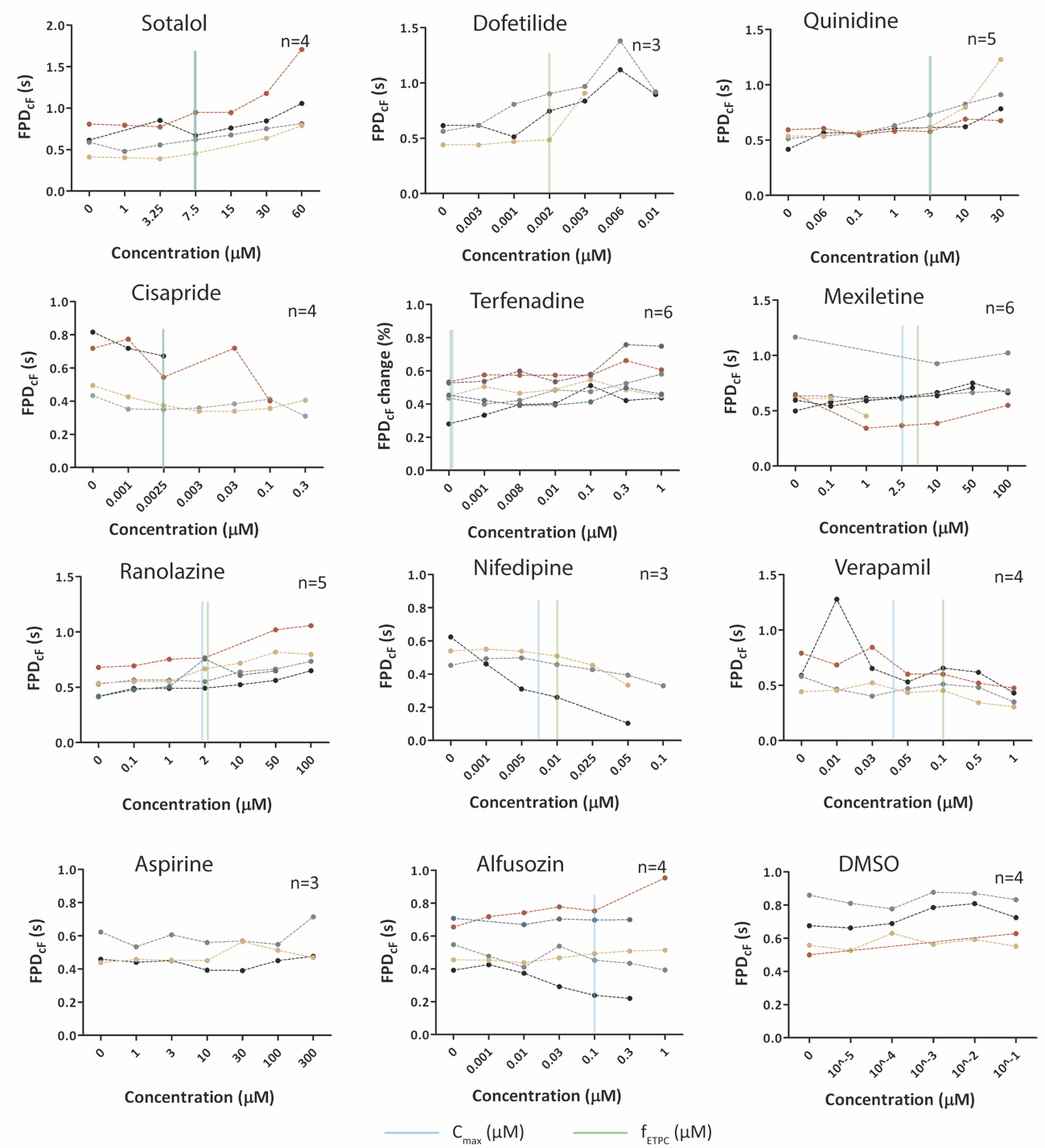


**Suppl Fig. 5**: FPD corrected with Fridericia formula of uHeart model subjected to different concentrations of drugs acting on single or multiple cardiac ion channels. For each drug, the C_max_ (vertical blue line) and the f_ETPC_ (vertical green line) are reported.

Supplementary Figure 6


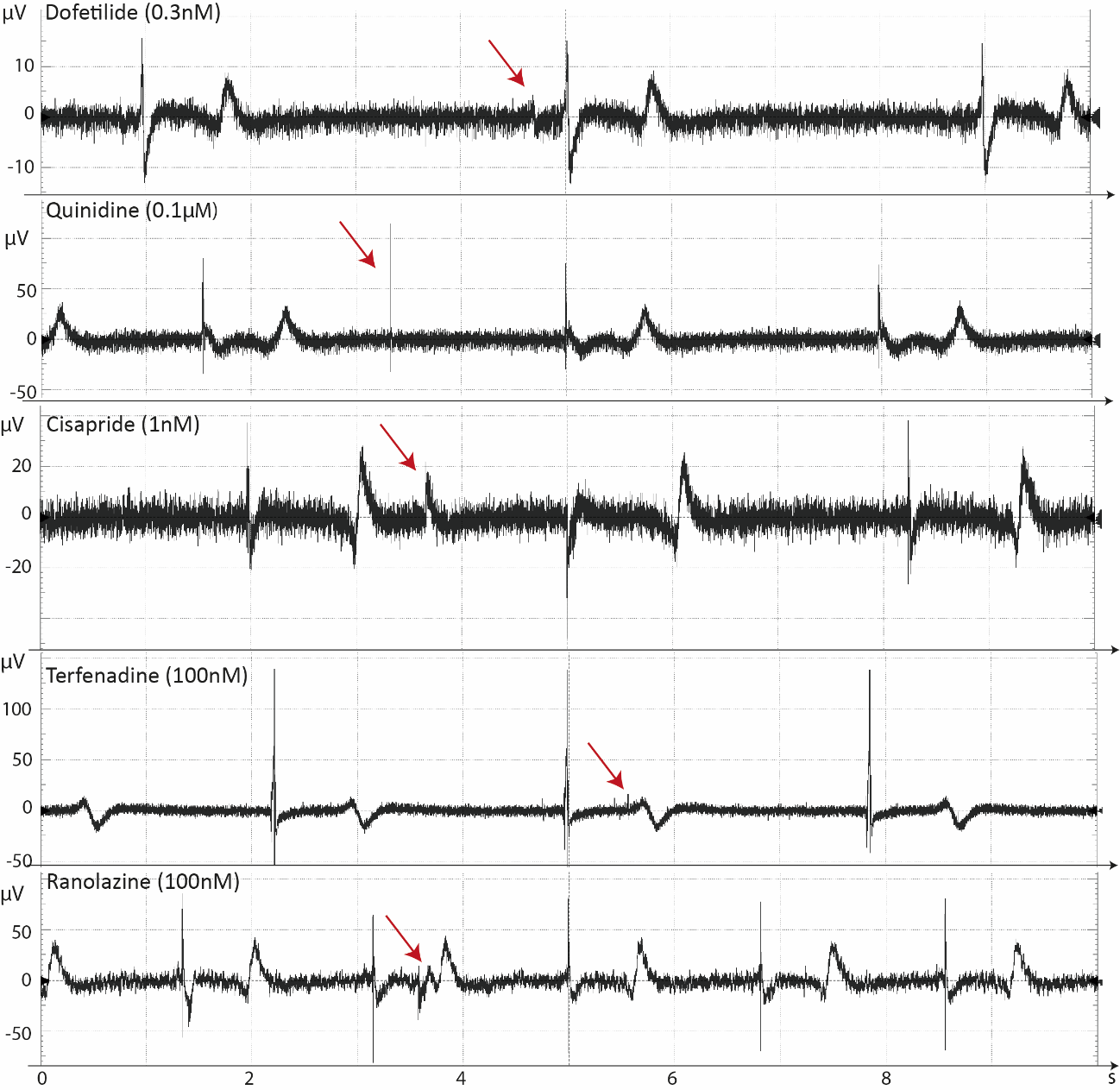


**Suppl Fig. 6**: Representative arrhythmic events recognized within the recorded signals of uHeart administered with Dofetilide, Quinidine, Cisapride, Terfenadine and Ranolazine.

Supplementary Figure 7


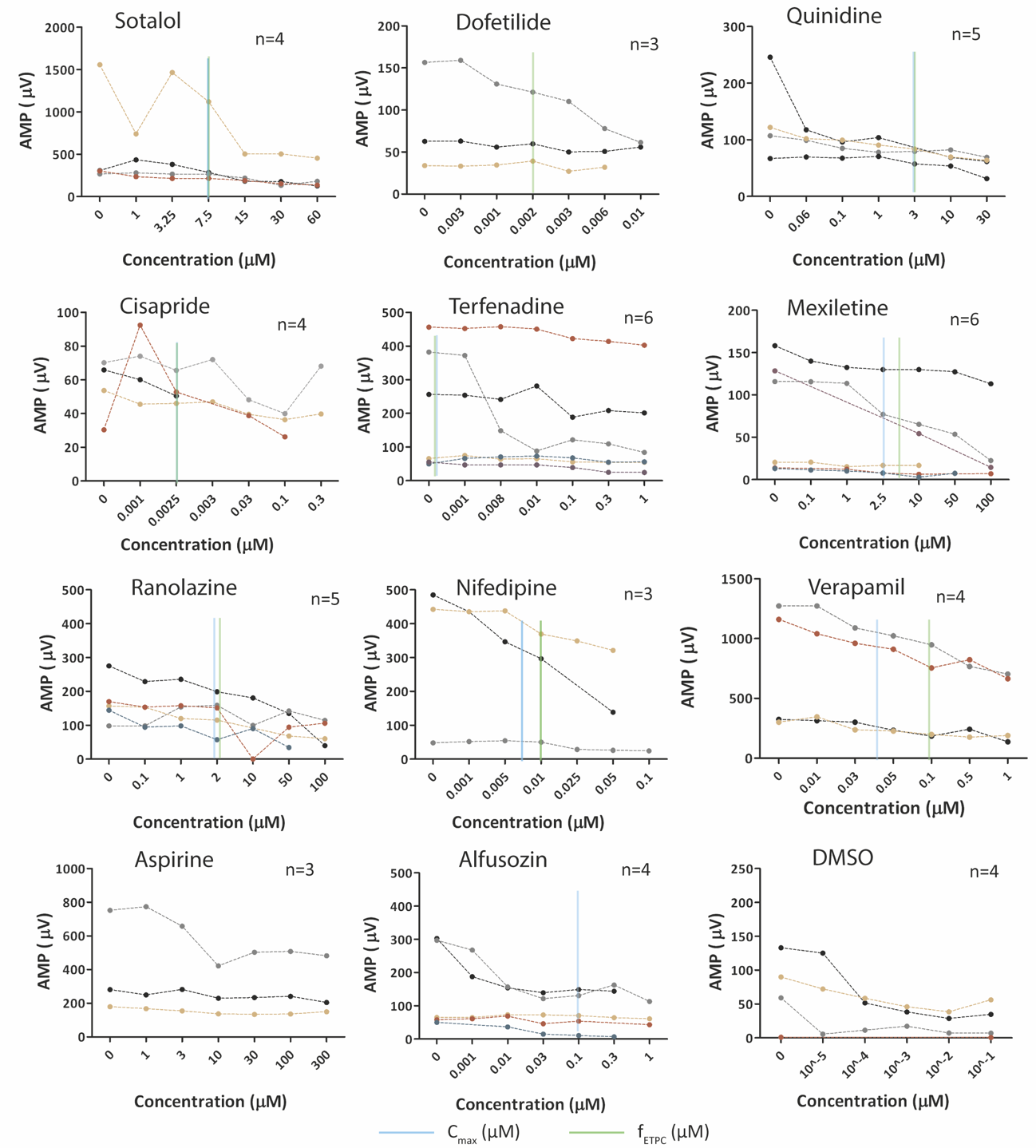


**Suppl Fig. 7**: Amplitude of uHeart’s field potentials when the model are subjected to different concentrations of drugs acting on single or multiple cardiac ion channels. For each drug, the C_max_ (vertical blue line) and the f_ETPC_ (vertical green line) are reported.

Supplementary Figure 8


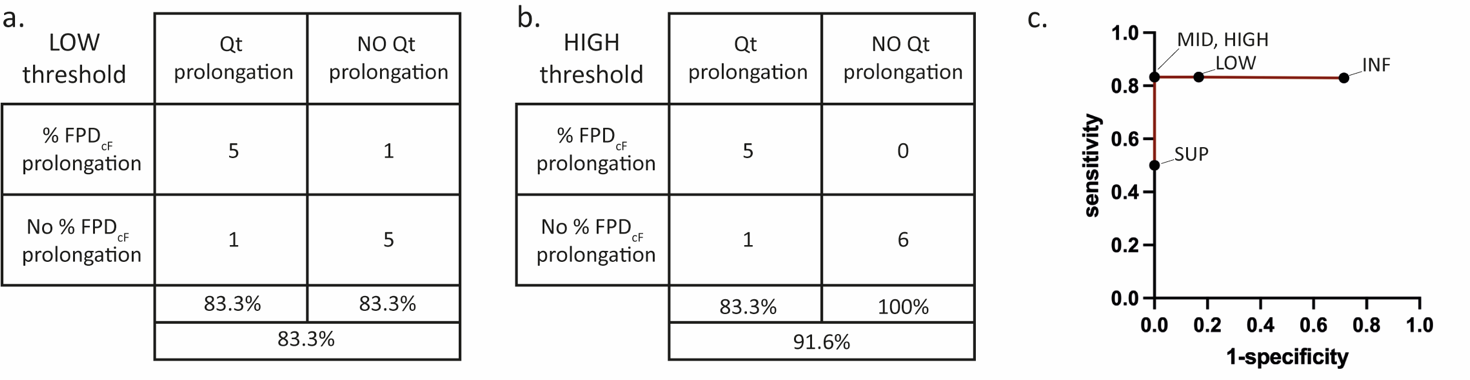


**Suppl Fig. 8**: Schematic representation of the concordance analyses and sensitivity, specificity and accuracy parameters performed by comparing the results obtained in uHeart with the FDA drug labels in case the threshold is a) LOW or b) HIGH. c) Receiver Operating Characteristic (ROC) curve describing the variation of sensitivity and specificity at the different threshold (i.e. LOW, MID, HIGH) as compared to two limit values (i.e. 5%-INF, 50%-SUP).
